# Dataset on physico-chemical characteristics of Exogenous Organic Matters (EOMs) gathered from various European countries

**DOI:** 10.1016/j.dib.2025.111585

**Published:** 2025-05-01

**Authors:** Aurélia Marcelline Michaud, Hélène Van Der Smissen, Lucille Caradec, Elina Tampio, Johanna Laakso, Florent Levavasseur, Karolina Barcauskaite, Donata Drapanauskaite, Maria Valentina Lasorella, Irene Criscuoli, Paulien Van Asperen, Janjo De Haan, Julie Jimenez, Sabine Houot

**Affiliations:** a INRAE, Institut Agro, UMR SAS, 35000, Rennes, France; b CRA-W, Department of Sustainability, Systems & Prospective – Unit of Soil, Water & Integrated Crop Production, Walloon Agricultural Research Centre (CRA-W), Rue du Bordia, 4, 5030 Gembloux, Belgium; c Natural Resources Institute Finland (Luke), Production Systems, Latokartanonkaari 9, 00790 Helsinki, Finland; d Université Paris-Saclay, INRAE, AgroParisTech, UMR ECOSYS, Palaiseau, France; e Lithuanian Research Centre for Agriculture and Forestry, Instituto al. 1, Akademija LT-58344 Kėdainiai, Lithuania; f CREA Research Centre for Agricultural Policies and Bioeconomy, Rome Italy; g Wageningen University & Research, Field Crops, Edelhertweg 1, 8219 PH, Lelystad, the Netherlands; h INRAE, Univ. Montpellier, LBE, 102 Avenue des étangs, 11100, Narbonne, France

**Keywords:** Biochar, Composition, Compost, Digestate, Livestock manure, Sludge, Urine

## Abstract

Many activities generate organic wastes, including urban activities (e.g., biowaste, sewage sludge), industry (e.g. vinasse) and agriculture (e.g., livestock manure, crop residues). Exogenous Organic Matters (EOMs) are secondary raw materials, i.e., wastes and residues from agriculture, municipalities or industry, which are either used as such or further processed with different technologies. The large variability in the raw materials and production technologies increases the diversity of EOM characteristics, which in turn affect their efficacy when applied to soils.

The datapaper presents the database “Physico-chemical characteristics of Exogenous Organic Matters (EOMs)” which is available in the Zenodo repository (https://doi.org/10.5281/zenodo.13969793). The database is a non-relational database in column format established in the framework of the EJP SOIL EOM4SOIL project, which aimed at establishing a database on EOM’s characteristics. The database gathered EOM characteristics collected in national databases and surveys from 6 European countries, and completed by data published in scientific articles. It describes physico-chemical characteristics of 126 types of EOMs encompassing urban, industrial and agricultural origins (e.g. urine, biowastes, sewage sludge, farmyard manures) and 91 characteristics (e.g. major elements, trace metals, emerging organic contaminants, pathogens, potentially mineralised C and N). There is an average of about 20 variables collected per type of EOM. Preliminary description of the EOM characteristics database is proposed in the present datapaper using descriptive statistics.

The characteristics of the 126 types of EOMs provide valuable insights that can help farmers, policymakers, and agricultural consultants to optimize the use of these materials in fertilization and soil amendment practices. This knowledge is essential for better management of EOM application practices by the farmers in order to increase soil carbon stocks and reduce the reliance on mineral fertilizers.

Specifications Table

**Table:** 

Subject	Earth & Environmental Sciences
Specific subject area	Physico-chemical properties of exogenous organic matters collected from national surveys and databases, and scientific publications
Type of data	Table CSV, XLS
Data collection	The database was created from the average composition of 126 EOM types from industrial, urban and agricultural origins. EOM characteristics were collected from six countries: Belgium, Finland, France, Italy, Lithuania and the Netherlands. Data originated from national inventories, national databases and national synthesis, depending on the availabilities of such studies in each country. In addition, data from literature have been added particularly for contaminants and pathogens. The following characteristics have been collected: physical parameters; concentrations of chemical parameters such as nutrients, trace metals and organic contaminants; pathogens; potential carbon and nitrogen mineralization during soil incubation; biochemical organic matter fractionation. Normalisation has been done by expressing all the concentrations on dry matter basis.
Data source location	INRAE internal databases, FRANCE
Data accessibility	Repository name: Zenodo Data identification number: none Direct URL to data: https://doi.org/10.5281/zenodo.13969793 Instructions for accessing these data: https://zenodo.org/records/14179949?token=eyJhbGciOiJIUzUxMiIsImlhdCI6MTc0MDU2NzgyNywiZXhwIjoxNzUxMjQxNTk5fQ.eyJpZCI6Ijk3OWIwYTJhLTdjNzAtNDc0ZC05OTZiLWYyM2QyOWMxMzcxOSIsImRhdGEiOnt9LCJyYW5kb20iOiJjOTZlMTllN2ZhZmU2ZjdlMmMzYTg5MmVhYTc4ZmMyZiJ9.-WLpUIFnhq9WGESTO8DHgSwRF3B5VRgv3hjs0ZkAp2gNejP_RRDk1HbdC7NCLhfBuUpph1U5_OQFlqMhpYWSTg
Related research article	None

## 1 Value of the Data

• This is the first time that a large dataset presents physico-chemical properties of such a diverse array of Exogenous Organic Matters (EOMs), with 126 processed and unprocessed EOMs from urban, industrial and agricultural origins, and from different European countries.
• This is the first time that a database includes such a wide diversity of agronomical properties of EOMs (e.g. pH, dry mass, macronutrient composition, micronutrients), pathogens and contaminants concentrations, including emerging contaminants (e.g. per and polyfluoroalkyl substances – PFAS, pharmaceutical residues) gathered from national surveys and databases, completed by peer-reviewed data.
• This data can be used to better comprehend the variability of EOMs properties generated by a range of raw materials types and treatment processes.
• The data is intended to be used as references by policymakers, agricultural researchers and advisors to more effectively plan the utilisation of these products as fertilizers and soil improvers in diverse climatic and soil conditions.
• The characteristics could be used for instance in the preparation of policy instruments and setting targets for both nutrient recycling and soil health.
• This database can be used to improve and parametrize models testing EOM inputs scenarios, multi-criteria and life cycle analysis studies, and fertilizing management tools.

## 2 Background

There is an increasing diversity of raw materials and technologies from which EOMs are produced. The characteristics of the raw material, the processing steps employed (e.g. anaerobic digestion, composting), and the parameters used (e.g., temperature, retention time, pH), all influence the quality and characteristics of the final product [1,2]. The large variability in the raw materials and production technologies increases the diversity of EOM characteristics, which in turn affect their efficacy when applied to soils [3].

Monitoring the use of EOMs, by characterising their agronomic value and by estimating soil contamination risks, will contribute to improved nutrient recycling, carbon return to soils, and soil health [3]. Within the EU, there are also national differences in the production processes of EOMs, products names and quality [4]. Accurate information on EOM nomenclature and related characteristics is needed to make appropriate national and European-level policy recommendations on EOMs utilisation. Previously published EOMs databases have focused on certain EOMs or characteristics [[5], [6], [7], [8]] but there are no studies focusing on a wide overview of characteristics for agronomical parameters, pathogens and contaminants for a large set of EOMs and origins.

## 3 Data Description

This article describes the “Physico-chemical characteristics of Exogenous Organic Matters” database of the linked repository containing characteristics from 126 EOM types encompassing urban, industrial and agricultural origins; and collected in six European countries: Belgium, Finland, France, Italy, Lithuania and The Netherlands. The repository contains three Excel files (also converted in .csv format), one containing physico-chemical characteristics, one describing the EOM nomenclature and one for the related metadata and controlled vocabularies defined in Thesaurus.

A user-friendly visualisation tool of EOM characteristics has been developed in R shiny to visualise the “Physico-chemical characteristics of Exogenous Organic Matters” database. The visualisation tool is available in Zenodo repository at: https://doi.org/10.5281/zenodo.14170693

**Fig. 1:**
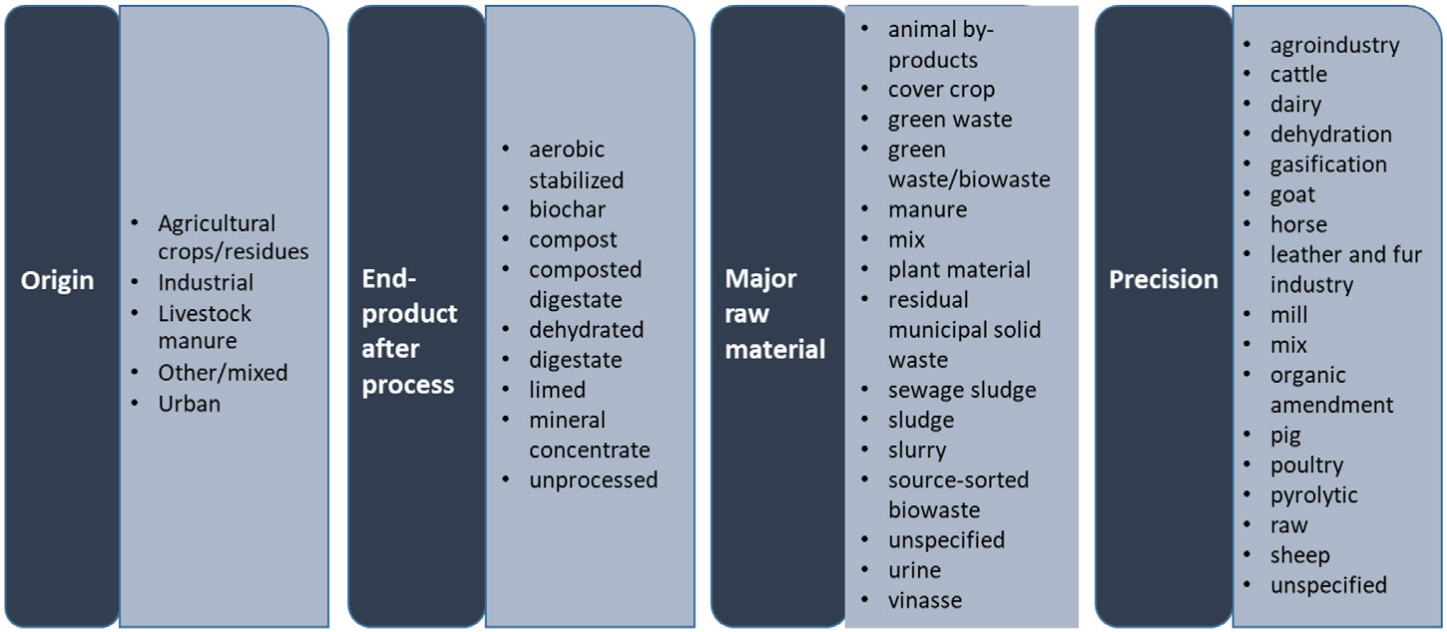
Sequential EOM nomenclature, with the following information presented: origin, end-product type after process, major raw material and precision.

The EOMs were described according to a proposed nomenclature of EOM denominations which was divided into four sub-categories (Fig. 1): (i) origin of raw material, (ii) end-product type after process, (iii) major raw material types, and (iv) additional information registered as “precision”. Similar nomenclature is also included in the Fertilising products regulation (2019/1009/EU) in the form of Component Material Categories (CMCs). CMCs define the allowed components of EU fertilising products and the thresholds for their characteristics. However, the categorization implemented in the present study is more detailed and enables the EOMs to be classified in the CMCs.

Considering the different countries, the “Physico-chemical characteristics of Exogenous Organic Matters” database gathered in total 126 EOMs according to the EOM nomenclature. For some EOMs, sub-types have been registered in order to better reflect the regionally generated EOMs (e.g. liquid and raw digestates, cattle manure from various origins).

**Fig. 2:**
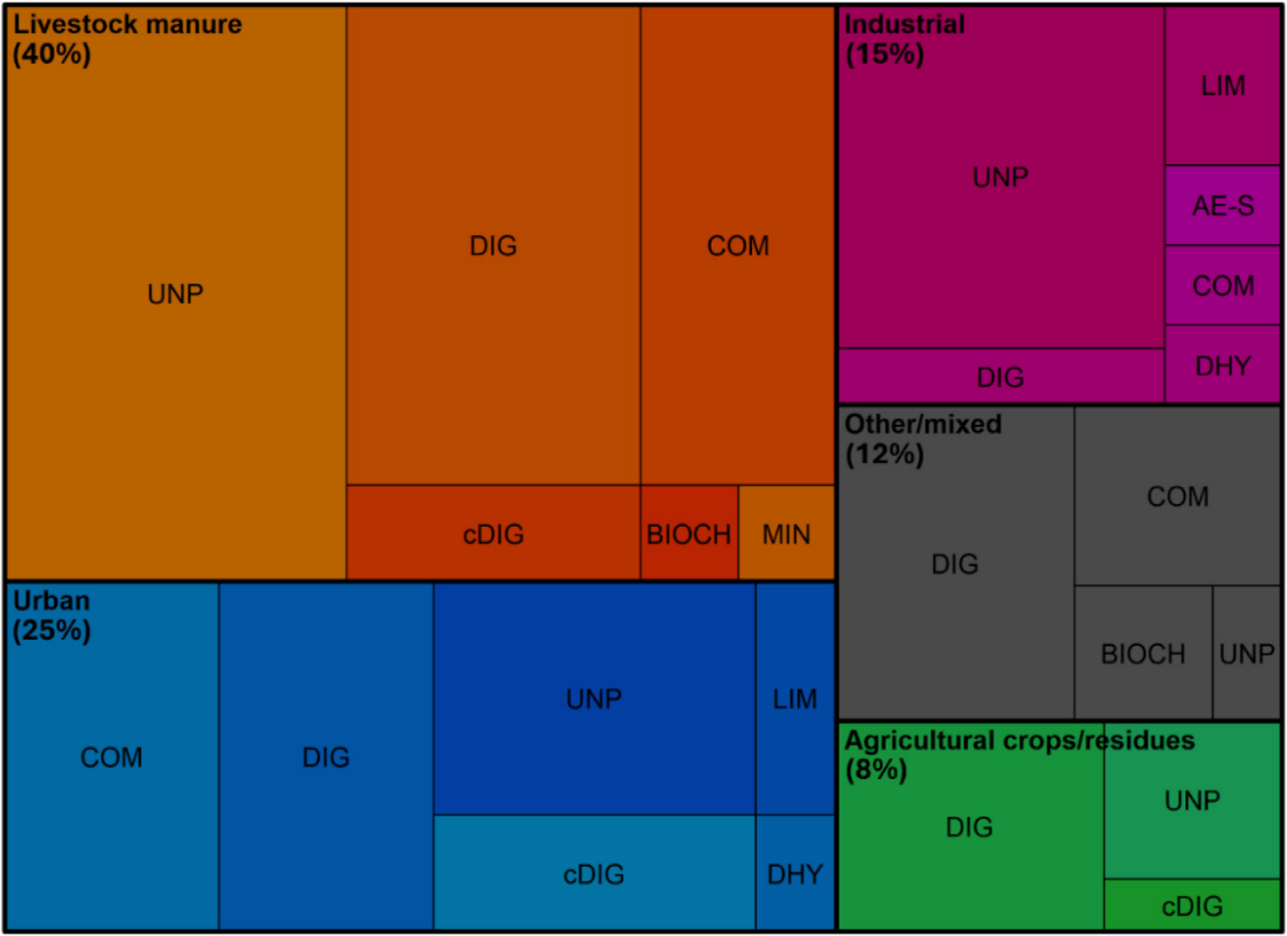
Treemap of the EOMs, with the partition of the different end-products after processes among origins. Acronyms: AE-S – Aerobically stabilized, BIOCH – biochar, COM – compost, cDIG – Composted digestate, DHY – Dehydrated, DIG – Digestate, LIM – Limed, MIN – Mineral concentrate, UNP – Unprocessed.

In the EOM database, 40% of the data refers to livestock manure EOMs, 25% to urban EOMs, 12% to other/mixed EOMs, 15% to industrial EOMs and 8% to agricultural crops/residues EOMs (Fig. 2). Considering the end-products after processing, 36% of the data concerned unprocessed EOMs, 31% digestates, 18% composts, 6% composted digestates, 3% limed, 2% dehydrated EOMs, 2% biochars, and less than 1% of the data concerned properties of aerobically stabilized EOMs and mineral concentrates. Most EOMs among livestock manure origin are unprocessed, followed by digestates and then composts (SI.1), with manures as major raw materials followed by slurries. EOMs of industrial origin are also mostly unprocessed. Urban raw materials are made up of different types of biowastes (municipal biowaste, sorted or unsorted, green waste) or sewage sludge, and one type is unprocessed urine. In the other/mix category, the raw materials composing digestates, composts or biochars are mixed raw materials, plant material, animal by-products or unspecified raw materials. Finally, in agricultural crops/residues origin, EOMs are composed of cover crops, plant material, mix or unspecified raw materials.

Overall, from the 126 EOMs, 91 physico-chemical characteristics were collected. The following main characteristics are presented with the percentage of detection across the 126 EOMs in brackets, and organised from the most frequent characteristic to the least frequent one: dry mass (DM, 100% of detection), total nitrogen (N_tot, 98%), total phosphorus (P_tot, 97%), total potassium (K_tot, 92%), organic matter (OM, 92%), ammonium nitrogen (N_NH4, 86%), pH (80%), carbon over nitrogen ratio (C/N, 70%), total copper (Cu, 61%), total zinc (Zn, 61%), total lead (Pb, 56%), total nickel (Ni, 56%), total cadmium (Cd, 55%), total chromium (Cr, 55%), total arsenic (As, 48%), total mercury (Hg, 44%), total sulphur (total_S, 31%), sum of 7 polychlorinated biphenyl (7 PCB, 25%) and sum of 16 polycyclic aromatic hydrocarbon (16 PAH, 24%). These main physico-chemical characteristics are described in the following Table 1, Table 2 with a statistical description per origin, with the percentage of detection, minimum, mean, maximum, standard deviation, quartiles and median. Additional descriptive analyses are presented in Supplementary information (including notably emerging contaminants such as PFAS and pharmaceutical residues; SI.2).

**Table 1:** Overview of the EOMs main agronomical characteristics, with the following data presented per origin: % (percentage) of EOM types with defined data, min (minimum), mean, max (maximum), SD (standard deviation), Q25 (first quartile), median, Q75 (third quartile).

Variable	Origin	%	Min.	Mean	Max.	SD	Q25	Median	Q75
Dry mass^1^	Crops/residues	100.0 %	3.0	16.2	50.0	14.9	5.5	9.7	21.2
	Manure	96.0 %	2.0	28.3	97.5	20.2	9.3	26.2	36.9
	Industrial	94.7 %	3.1	36.2	98.9	26.4	16.1	31.2	53.0
	Urban	93.6 %	1.1	41.6	94.3	25.1	19.7	45.9	60.9
	Other/mixed	86.7 %	5.7	30.8	92.9	28.2	7.5	25.3	46.0
pH	Crops/residues	70.0 %	7.3	8.0	8.7	0.4	7.9	8.0	8.2
	Manure	66.0 %	5.9	8.2	9.4	0.7	7.9	8.1	8.6
	Industrial	89.5 %	6.0	8.6	12.8	1.9	7.5	8.1	9.5
	Urban	83.9 %	6.8	8.3	12.5	1.2	7.7	8.1	8.5
	Other/mixed	86.7 %	7.3	8.5	10.6	0.8	8.1	8.3	8.7
C/N	Crops/residues	70.0 %	2.8	7.0	13.6	3.3	4.8	6.2	8.8
	Manure	68.0 %	2.9	11.4	34.3	7.1	6.5	10.1	13.4
	Industrial	79.0 %	3.7	14.8	38.2	10.8	6.0	13.0	18.0
	Urban	61.3 %	0.7	9.6	19.1	4.9	5.6	10.3	13.3
	Other/mixed	66.7 %	3.9	42.9	388.6	100.3	4.6	8.1	17.3
N_total^²^	Crops/residues	100.0 %	7.2	64.8	160.0	44.6	31.7	60.6	77.1
	Manure	94.0 %	8.0	47.8	325.0	46.1	25.6	33.0	54.1
	Industrial	94.7 %	0.8	25.7	133.3	35.3	4.7	9.4	27.8
	Urban	90.3 %	6.7	52.6	536.4	86.8	16.5	22.7	55.8
	Other/mixed	100.0 %	1.8	44.4	105.0	36.5	14.8	24.4	71.4
N_NH4^²^	Crops/residues	90.0 %	0.0	39.1	82.7	30.1	8.2	39.6	67.4
	Manure	82.0 %	0.1	18.3	112.1	22.4	3.7	8.8	26.1
	Industrial	73.7 %	0.0	12.1	104.2	28.7	0.1	0.2	4.7
	Urban	87.1 %	0.0	25.7	463.6	76.6	1.2	4.8	18.6
	Other/mixed	73.3 %	0.9	22.1	67.8	20.6	5.5	17.8	30.6
K_total^²^	Crops/residues	90.0 %	2.6	43.6	94.6	32.2	13.1	42.7	72.3
	Manure	88.0 %	9.3	41.3	266.0	41.6	21.9	30.6	45.7
	Industrial	89.5 %	0.2	14.3	82.8	26.9	0.6	1.2	9.0
	Urban	87.1 %	1.2	16.9	154.5	28.3	4.6	8.6	12.8
	Other/mixed	93.3 %	2.6	31.9	80.2	23.6	14.4	28.0	50.9
Org. matter^²^	Crop/residues	100.0 %	103	603	800	197	552	659	724
	Industrial	78.9 %	129	433	887	257	181	420	622
	Manures	90.0 %	250	691	912	129	627	686	779
	Other/mixed	75.0 %	44	572	771	178	564	621	651
	Urban	87.1 %	299	522	780	120	428	501	616
P_total²	Crops/residues	90.0 %	1.6	14.7	47.5	12.2	8.9	9.9	17.5
	Manure	94.0 %	2.0	11.4	41.0	7.1	6.9	10.0	14.6
	Industrial	94.7 %	0.5	10.3	42.2	12.6	1.6	4.0	17.1
	Urban	90.3 %	1.4	12.4	36.2	9.7	4.2	10.5	16.8
	Other/mixed	93.3 %	0.3	15.4	34.0	9.6	8.7	15.8	19.9
S_total^²^	Crops/residues	20.0 %	2.7	3.6	4.5	1.3	3.1	3.6	4.0
	Manure	50.0 %	0.6	9.0	39.0	10.7	3.8	5.3	8.0
	Industrial	10.5 %	5.6	9.9	15.0	4.8	7.3	9.0	12.0
	Urban	9.7 %	5.0	5.9	7.0	1.0	5.3	5.6	6.3
	Other/mixed	40.0 %	0.2	5.8	8.4	2.6	5.3	6.4	7.2

Notes: ^1^expressed in % of fresh matter, ²expressed in gram per kilogram of dry matter.

**Table 2:** Overview of the EOMs main trace metals and organic contaminants concentrations, with the following data presented per origin: % (percentage) of EOM types with defined data, min (minimum), mean, max (maximum), SD (standard deviation), Q25 (first quartile), median, Q75 (third quartile); expressed in milligram per kilogram of dry matter.

Variable	Origin	%	Min.	Mean	Max.	SD	Q25	Median	Q75
As_total	Crops/residues	40 %	0.8	1.8	3.4	1.2	0.9	1.2	2.9
	Manure	40 %	0.0	1.0	4.4	1.0	0.3	0.7	1.5
	Industrial	68.42 %	0.1	1.9	4.9	1.3	1.2	1.8	2.1
	Urban	48.39 %	2.1	5.5	12.7	2.9	2.8	5.0	7.4
	Other/mixed	46.67 %	0.0	2.5	8.4	3.3	0.7	0.9	2.1
Cd_total	Crops/residues	40.0 %	0.3	0.5	0.7	0.1	0.5	0.5	0.6
	Manure	52.0 %	0.0	0.4	0.9	0.2	0.2	0.4	0.5
	Industrial	68.4 %	0.1	0.6	1.8	0.5	0.2	0.5	0.9
	Urban	61.3 %	0.2	1.0	4.6	0.9	0.6	0.9	1.1
	Other/mixed	53.3 %	0.3	0.6	1.0	0.2	0.5	0.7	0.8
Cr_total	Crops/residues	40.0 %	2.1	15.5	26.1	8.8	13.9	16.6	19.0
	Manure	54.0 %	0.3	7.8	24.7	5.4	4.7	6.2	9.6
	Industrial	68.4 %	1.7	31.6	268.0	68.6	8.7	12.3	20.4
	Urban	61.3 %	3.2	35.0	126.3	25.7	18.7	30.0	37.5
	Other/mixed	46.7 %	4.2	25.7	75.0	19.7	14.5	17.0	28.9
Cu_total	Crops/residues	40.0 %	57.2	130.6	217.6	59.2	105.7	122.7	149.7
	Manure	66.0 %	2.0	99.5	486.0	102.9	33.3	71.8	120.0
	Industrial	68.4 %	9.2	45.2	158.6	42.2	22.6	27.4	52.4
	Urban	61.3 %	12.0	134.8	384.7	122.3	46.1	82.9	186.3
	Other/mixed	60.0 %	9.7	78.7	172.6	55.8	25.5	72.0	117.5
Hg_total	Crops/residues	20.0 %	0.0	0.3	0.6	0.4	0.2	0.3	0.4
	Manure	30. %	0.0	0.2	2.7	0.5	0.0	0.1	0.1
	Industrial	68.4 %	0.0	0.2	1.2	0.4	0.0	0.1	0.1
	Urban	54.8 %	0.0	0.4	1.9	0.5	0.1	0.2	0.6
	Other/mixed	46.7 %	0.0	0.2	0.4	0.1	0.1	0.1	0.2
Ni_total	Crops/residues	40.0 %	9.4	12.4	15.4	2.2	11.9	11.9	13.2
	Manure	54.0 %	0.7	15.8	154.6	22.8	6.2	9.5	17.4
	Industrial	68.4 %	4.6	13.1	33.0	7.6	6.6	12.5	15.8
	Urban	61.3 %	3.2	20.7	60.4	11.4	12.3	20.4	25.5
	Other/mixed	60.0 %	5.9	13.2	22.5	5.2	9.2	13.5	15.9
Pb_total	Crops/residues	40.0 %	7.4	9.0	12.0	1.7	8.4	8.5	8.7
	Manure	54.0 %	0.1	4.5	34.2	6.4	0.9	2.6	4.9
	Industrial	68.4 %	1.0	7.6	18.8	6.0	3.1	5.5	13.4
	Urban	61.3 %	2.5	59.4	352.9	62.6	32.1	44.9	63.5
	Other/mixed	60.0 %	1.5	12.9	35.8	10.1	8.2	10.9	13.1
Zn_total	Crops/residues	40.0 %	186.3	407.1	696.6	223.6	211.5	367.7	573.6
	Manure	66.0 %	12.0	407.1	4050.0	600.5	142.0	203.2	400.8
	Industrial	68.4 %	22.8	182.8	774.7	196.1	46.2	137.0	220.7
	Urban	61.3 %	63.0	391.7	994.9	252.3	177.4	325.7	582.6
	Other/mixed	60.0 %	21.5	279.2	524.1	165.3	153.9	286.8	393.4
16 PAH	Manure	12.0 %	0.2	3.3	16.5	5.4	0.6	1.6	2.4
	Industrial	52.6 %	0.1	0.6	1.9	0.5	0.2	0.3	0.8
	Urban	38.7 %	0.9	3.5	16.9	4.3	1.5	2.5	3.3
	Other/mixed	26.7 %	0.5	1.3	2.3	0.7	1.0	1.0	1.9
7 PCB	Manure	10.0 %	0.0	0.0	0.1	0.0	0.0	0.0	0.1
	Industrial	52.6 %	0.0	0.2	1.5	0.5	0.0	0.0	0.1
	Urban	45.2 %	0.0	0.1	0.4	0.1	0.0	0.0	0.1
	Other/mixed	20.0 %	0.0	0.1	0.1	0.0	0.0	0.1	0.1

Table 1 presents the main agronomical characteristics of EOMs from different origins. EOMs from urban and industrial origins exhibit the highest DM, with median values of 45.9% and 31.2%, respectively. Average N concentration is between 25.7 and 64.8 g/kg DM across origins, with some extreme values, such as a maximum of 536.4 g/kg for some urban EOMs. K concentration was higher in EOMs from agricultural origins (i.e. livestock manure and crops/residues) compared to EOMs from other origins, while P concentration was slightly larger in crops/residues and other/mixed origins compared to the others. In average, S concentration was higher in EOMs from livestock manure and industrial origins (9.0-9.9 g/kg DM) compared to the other origins in which its average concentration went from 3.6 g/kg DM in crops/residues to 5.8/5.9 g/kg DM in urban and other/mixed origins. Average pH values differed from 8.0 to 8.6 among EOM origins, while average C/N ratios differed widely among origins with larger values in other/mixed origin and lower values for crops/residues and urban origins.

Table 2 presents the concentrations of total trace metals and organic contaminants in EOMs. Urban EOMs contain globally the highest concentrations of trace metals and organic contaminants, having the highest third quartile of 7.4 mg/kg DM for As, 1.1 mg/kg DM for Cd, 37.5 mg/kg DM for Cr, 186.3 mg/kg DM for Cu, 0.6 mg/kg DM for Hg, 25.5 mg/kg DM for Ni, 63.5 mg/kg DM for Pb, 582.6 mg/kg DM for Zn and 3.3 mg/kg DM for 16 PAH. Livestock manure origin shows lower levels of most trace metals except for Cu and Zn, with a third quartile of 120.0 mg/kg DM and 400.8 mg/kg DM, respectively. EOMs originating from crops/residues generally exhibit among the lowest contaminant levels except for Cu and Zn, while other/mixed EOMs show intermediate levels, with substantial variability depending on the contaminants. In addition, contaminant concentrations displayed variability within the same EOM origin, especially for Cu, Ni, Zn and 16 PAH in livestock manure EOMs; Cd, Pb and 16 PAH in urban EOMs; Cr in industrial EOMs; Cu and Zn in crops/residues EOMs.

Whatever the EOM origin was, the averages, medians and third quartiles for all trace metals concentrations were below the European thresholds established for organic fertilizers and soil amendments in the European regulation (EU 2019/1009) (Tables 2, 3). When considering maximal concentrations, some overpassed EU regulation thresholds for organic fertilizer and amendment: Cu, Hg, Ni and Zn in livestock manure and urban EOMs, Pb in urban EOMs, Hg in industrial EOMs, Cd in industrial and urban EOMs.

**Table 3:** Thresholds mentioned in European regulations (Fertilizing products regulation - EU 2019/1009, Product Function Categories - PFC) for trace metals concentrations expressed in mg/kg of dry matter.

	Organic fertilizer - EU 2019/1009 - PFC1-A	Organic soil amendment - EU 2019/1009 - PFC3-A
As	40	40
Cd	1.5	2
Cr tot	-	-
CrVI	2	2
Cu	300	300
Hg	1	1
Ni	50	50
Pb	120	120
Zn	800	800

**Fig. 3:**
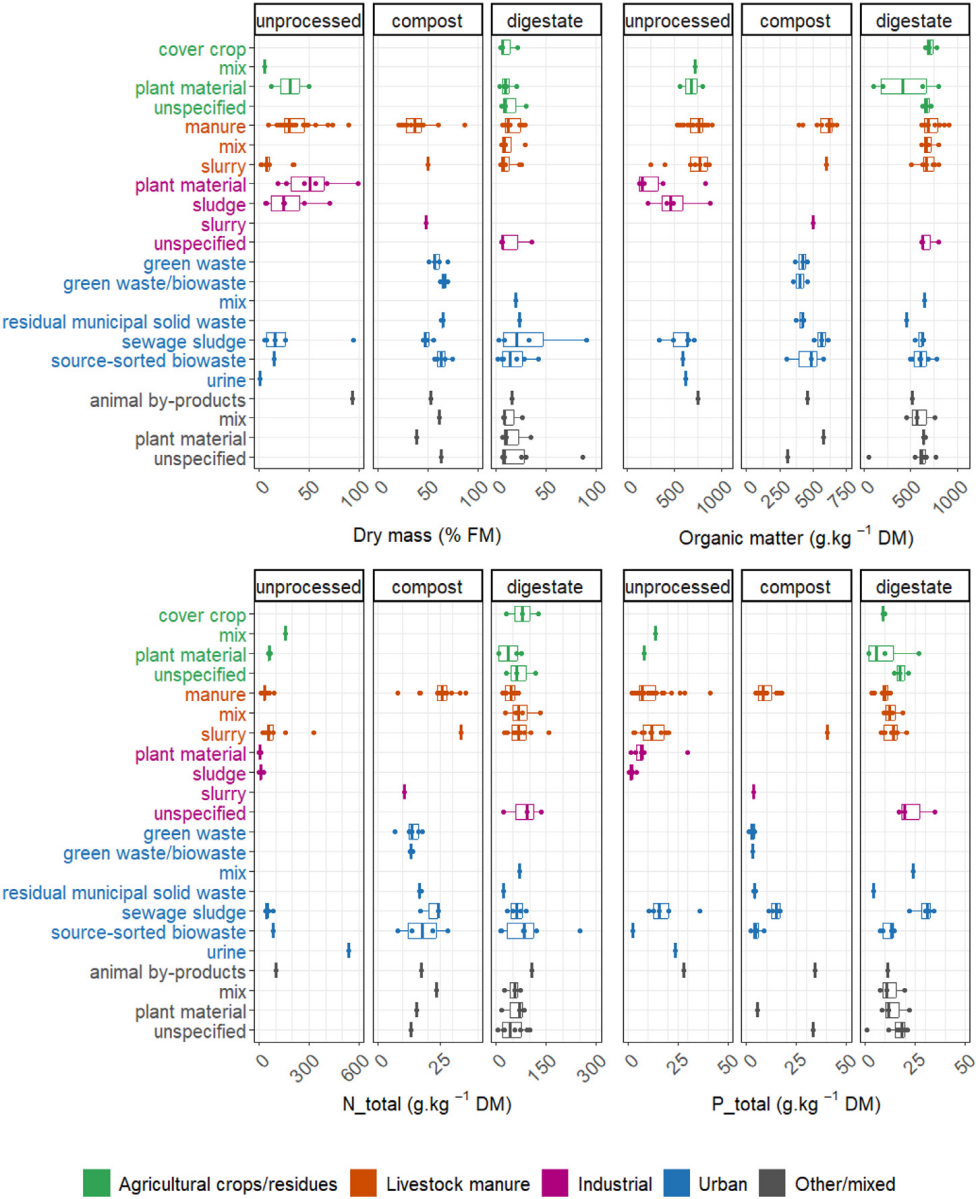
Box plots for selected major variables for nutrient composition (dry mass, organic matter, total nitrogen and phosphorus) according to the EOMs nomenclature, i.e. origin (agricultural crops/residues, livestock manure, industrial, urban and other/mixed origins), 3 main end-products after process (i.e. unprocessed, compost and digestates) and related major raw materials.

**Fig. 4:**
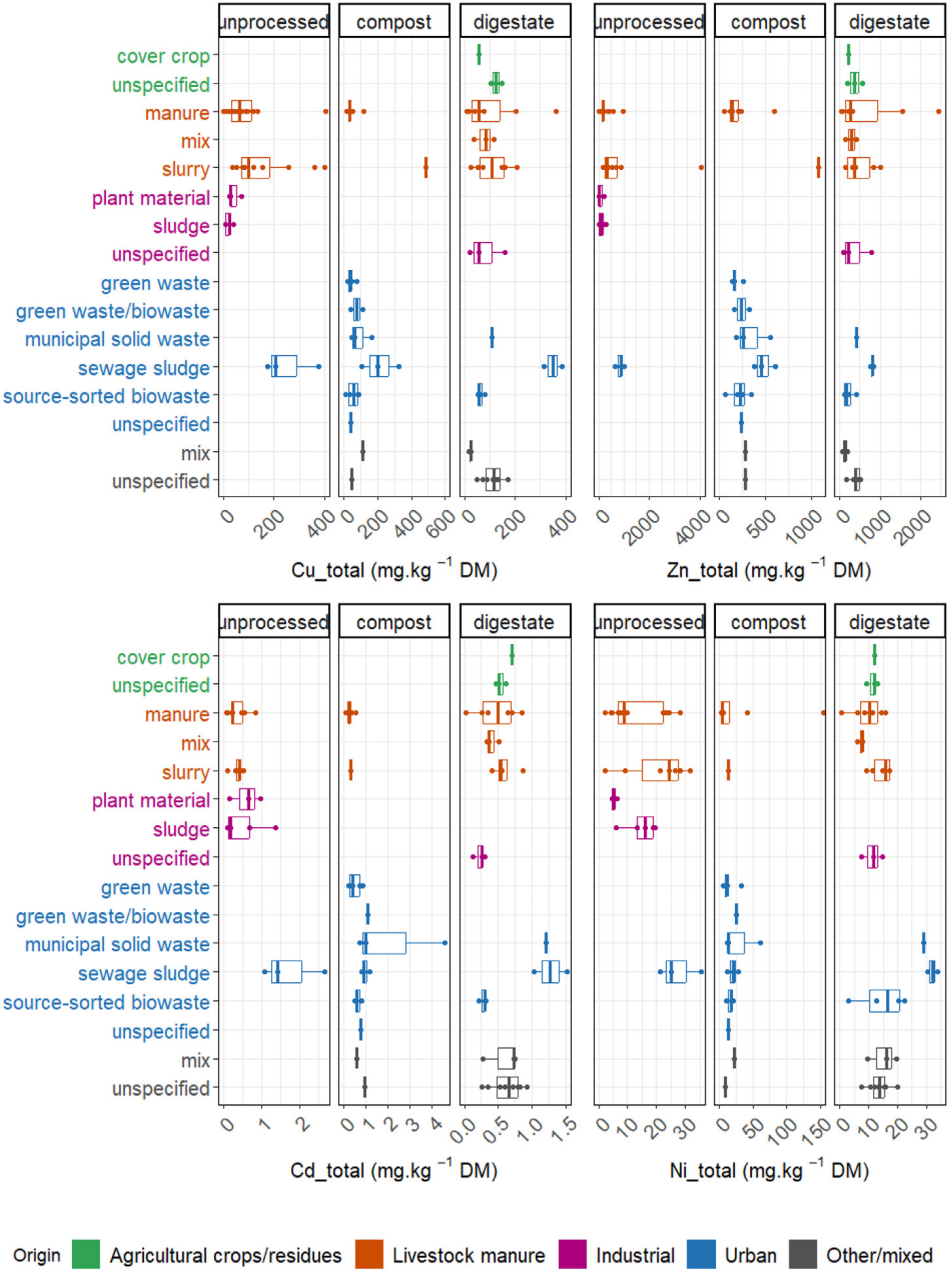
Box plots for selected major variables for trace metals (copper, zinc, cadmium and nickel), according to the EOMs nomenclature, i.e. origin (agricultural crops/residues, livestock manure, industrial, urban and other/mixed origins), 3 main end-products after process (i.e. unprocessed, compost and digestates) and related major raw materials.

Given the variability of EOMs characteristics depending on their origin, in Fig. 3, Fig. 4 we explore the characteristics variation for selected major variables according to the EOMs nomenclature, i.e. the origin, end-products after processing by considering the main ones (i.e. unprocessed, compost and digestate) and the related major raw materials. Fig. 3 shows the box plot for DM, OM, total N and P concentrations, while Fig. 4 presents box plots for Cu, Zn, Cd and Ni concentrations. Some EOMs were not reported as they do not represent end-products used in agriculture, e.g. urban unprocessed green waste - biowaste - municipal solid waste. There were some disparities between EOMs for some characteristics due to the putative limited availability of data, as some characteristics were missing for some EOMs (e.g. phosphorus in industrial compost produced with mixed raw materials) (Fig. 3).

DM values displayed high heterogeneity for unprocessed EOMs, with median values ranging from 1.1 to 92.9% of fresh matter (FM), while it was more homogeneous in digestates (6.5 to 23.3% of FM) and composts (37.0 to 66.0% of FM) (Fig. 3). Similarly, OM concentration displayed variability for unprocessed EOMs (median varying from 157.0 to 772.0 g/kg DM), while it was less variable for digestates (416.5 to 700.3 g/kg DM) and composts (402.5 to 617.7 g/kg DM). N concentration varied according to the end-product and raw material natures. Composts exhibited lower N concentration (median varying from 10.6 to 33.5 g/kg DM) compared to unprocessed end-products and digestates, with urine, digestate of animal by-products and urban digestate of source-sorted biowastes having the highest median N concentration (536.4, 105.0 and 83.2 g/kg DM, respectively). P concentration presented some variability depending on the end-product nature with larger variability observed in unprocessed EOMs and digestates, compared to composts. The highest P concentration was observed in urban digestates of sewage sludge, composts of livestock slurry and compost of animal by-products with a median concentration of 31.1, 40.4 and 34.0 g/kg DM, respectively.

As observed in Fig. 4, Cu concentration varied according to the EOM origin and major raw material nature, with the largest values observed in EOMs composed in majority by livestock slurry (unprocessed and compost) and urban sewage sludge (unprocessed, compost and digestate). The threshold for organic fertilizers and amendments set by the EU regulation 2019/1009 (300 mg/kg DM, Table 3) was exceeded in the case of livestock slurry compost and urban digestate of sewage sludge. Average Zn concentration in EOMs was lower than the EU regulation thresholds (800 mg/kg DM), except for livestock slurry compost and urban digestate of sewage sludge. Sewage sludge is not considered in the EU 2019/1009 regulation but is under load by the Directive 86/278/CEE in which Zn limit is 2000 mg/kg DM. Cd concentration in EOMs was lower than the EU regulation threshold of 1.5 mg/kg DM for organic fertilizers (Table 3), except for urban sewage sludge and to some extent for urban compost of municipal solid wastes. Nevertheless, all digestates, even when produced with those 2 major raw materials exhibited Cd levels lower than the threshold of 1.5 mg/kg DM. Globally, Ni concentration did not exceed the threshold level of 50 mg/kg DM mentioned in regulations. The highest levels were observed in EOMs composed in majority by sewage sludge and municipal solid wastes. Among urban EOMs, those ones composed in majority with biowastes and green wastes exhibited the lowest trace elements concentrations, i.e. under or in the same order of magnitude of the threshold mentioned in regulations (Table 3).

## 4 Experimental Design, Materials and Methods

In the framework of the EJP SOIL EOM4SOIL project, an Excel-based data collection template was generated in collaboration with partners to standardise and collect the denomination and the characteristics of EOMs across 6 European countries (SI.3, SI.4). Beyond facilitating harmonisation across countries, this format allowed efficient data storage and reuse.

As mentioned previously, a generic nomenclature system was developed to describe all types of EOMs based on 4 key criteria: origin, end-product after process, major raw material, and further precision (Fig. 1). This flexible system accommodates varying levels of details and allows the inclusion of EOMs with incomplete description. This nomenclature of EOMs integrated the diversity of EOMs produced and used in agriculture among EU countries, with the aim to harmonise EOM denomination description and filtering, and to collect organized information.

In addition to the EOM nomenclature, the physico-chemical characteristics were recorded based on same metadata lists to facilitate further data pooling and statistical analysis (See metadata in Zenodo repository). This includes (a) variables with agronomical characteristics (dry mass, carbon, nitrogen, phosphorus, potassium, …), contaminants (trace metals, organic contaminants including emerging ones), pathogens, potentially mineralised C and N and further indexes, (b) analytical methods depending on variables, (c) units and (d) related humidity fixed per variable. Such harmonised lists of metadata were created from the expertise of the French observatory devoted to the recycling of organic waste products [9,10] with the improvement and validation of the EOM4SOIL partners.

Finally, the template allowed to fill for each EOM of the nomenclature, the studied variables, the related analytical method, unit and humidity, mean, minimum and maximum values, standard deviation, number of samples considered, adding information and the source of the data. All data was encoded in consistent units, determined by the data collector to ensure uniformity regardless of the data source. To collect physico-chemical characteristics data of EOMs, the Excel spreadsheet was distributed to partners of the EOM4SOIL consortium across ten countries for data entry. Accurate replies from 6 partners were received, as indicated in the map in SI.4 for Belgium, Finland, France, Italy, Lithuania and the Netherlands. Data originated from national inventories, national databases (e.g. French FertiDig project for digestates) and national synthesis, depending on the availability of such sources in each partner country. Data from scientific publications completed the national datasets for some contaminants and pathogens. Once data was collected, a harmonisation and validation process was undertaken to ensure consistency and comparability between the different datasets. The validated non-relational database (in column format) describing the EOMs and related physico-chemical characteristics gathered from 6 countries was added to the Zenodo repository.

## Limitations

The data collection and pooling from six countries revealed disparities between countries and EOMs in terms of physico-chemical characteristics. The principal reasons for disparities come from the lack of European standards to name EOMs and to measure their characteristics, and from the limited availability of synthesis studies, inventories and national databases. In addition, there were discrepancies in the data quality and quantity, notably related to different standards existing at national levels but not at European level.

This work can be seen as the first step in initiating an EU-level EOM nomenclature and physico-chemical characterisation, as similar work has not been executed before. Nevertheless, such detailed knowledge and database describing EU-level EOM composition with systematic EOM classification based on raw materials and processing, would be an essential tool in decision making.

## Ethics Statement

The authors have read and followed the ethical requirements for publication in Data in Brief and confirm that the current work does not involve human subjects, animal experiments, or any data collected from social media platforms.

## CRediT Author Statement

**Aurélia Marcelline Michaud:** Conceptualization, Methodology, Validation, Investigation, Resources, Data Curation, Writing - Original Draft, Supervision, Project administration, Funding acquisition; **Hélène Van Der Smissen:** Validation, Formal analysis, Investigation, Resources, Data Curation, Writing - Original Draft; **Lucille Caradec:** Validation, Formal analysis, Investigation, Resources, Data Curation, Writing - Original Draft; **Elina Tampio:** Conceptualization, Methodology, Validation, Investigation, Resources, Data Curation, Writing - Review & Editing, Funding acquisition; **Johanna Laakso:** Conceptualization, Methodology, Validation, Investigation, Resources, Data Curation, Writing - Review & Editing; **Florent Levavasseur:** Resources, Validation, Data Curation; **Karolina Barcauskaite:** Resources, Validation, Data Curation, Funding acquisition; **Donata Drapanauskaite:** Resources, Validation, Data Curation; **Maria Valentina Lasorella:** Resources, Validation, Data Curation; **Irene Criscuoli:** Resources, Validation, Data Curation; **Paulien Van Asperen:** Resources, Validation, Data Curation; **Janjo De Haan:** Resources, Validation, Data Curation; **Julie Jimenez:** Resources, Validation, Data Curation, Writing - Review & Editing; **Sabine Houot:** Resources, Validation, Writing - Review & Editing, Project administration, Funding acquisition.

## Data Availability

ZenodoEOM4SOIL - Physico-chemical characteristics of external organic matters (EOMs) database (Reference data).

## Appendix Supplementary materials

Image, application 1
