# Supplementary material for: Dataset on physico-chemical characteristics of Exogenous Organic Matters (EOMs) gathered from various European countries
Source: Data Brief. 2025 May 1;60:111585. doi: 10.1016/j.dib.2025.111585 (PMC12138917; doi:10.1016/j.dib.2025.111585)
Supplement: Supplementary file 1 [file mmc1.docx]

Supplementary Information

**Dataset on physico-chemical characteristics of exogenous organic matters (EOMs) gathered from various European countries**

**Authors**

Michaud Aurélia Marcelline* [a], Van Der Smissen Hélène [b], Caradec Lucille [a], Tampio Elina [c], Laakso Johanna [c], Levavasseur Florent [d], Barcauskaite Karolina [e], Drapanauskaite Donata [e], Lasorella Maria Valentina [f], Criscuoli Irene [f], Van Asperen Paulien [g], De Haan Janjo [g], Jimenez Julie [h], Houot Sabine [d]

**Affiliations**

[a] INRAE, Institut Agro, UMR SAS, 35000, Rennes, France
[b] CRA-W, Department of Sustainability, Systems & Prospective – Unit of Soil, Water & Integrated Crop Production, Walloon Agricultural Research Centre (CRA-W), Rue du Bordia, 4, 5030 Gembloux, Belgium
[c] Natural Resources Institute Finland (Luke), Production Systems, Latokartanonkaari 9, 00790 Helsinki, Finland
[d] Université Paris-Saclay, INRAE, AgroParisTech, UMR ECOSYS, Palaiseau, France
[e] Lithuanian Research Centre for Agriculture and Forestry, Instituto al. 1, Akademija LT-58344 Kėdainiai, Lithuania
[f] CREA Research Centre for Agricultural Policies and Bioeconomy, Rome Italy
[g] Wageningen University & Research, Field Crops, Edelhertweg 1, 8219 PH, Lelystad, The Netherlands
[h] INRAE, Univ. Montpellier, LBE, 102 Avenue des étangs, 11100, Narbonne, France

SI.1. Treemap of the EOM types, with the partition of the different major raw materials grouped by the resulting end-products after processes among origins.


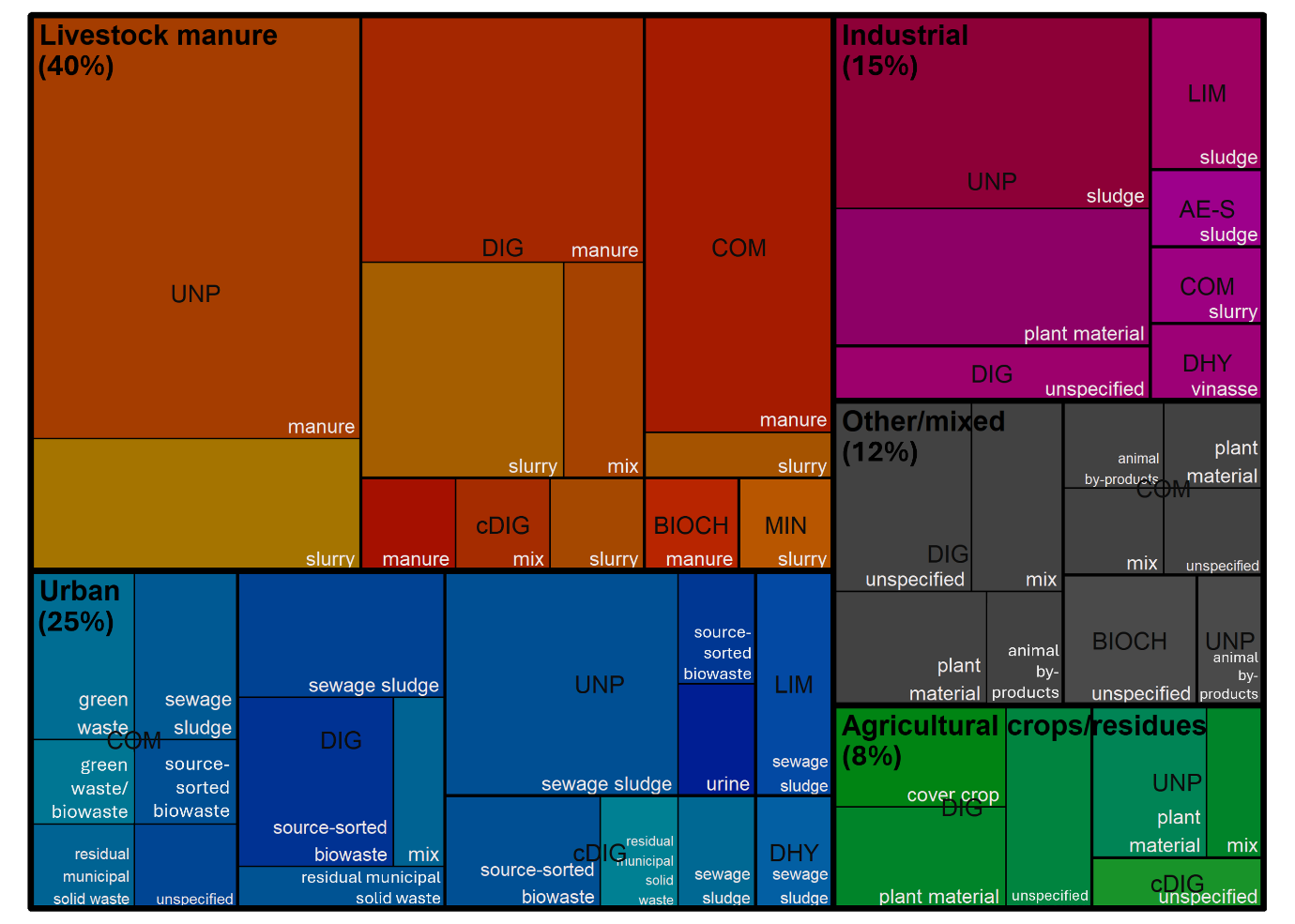


Acronyms: AE-S – Aerobically stabilized, BIOCH – biochar, COM – compost, cDIG – Composted digestate, DHY – Dehydrated, DIG – Digestate, LIM – Limed, MIN – Mineral concentrate, UNP – Unprocessed.

SI.2. EOM properties overview, for the complete database, with the following data presented: % (percentage) of detection, min (minimum), mean, max (maximum), SD (standard deviation), Q25 (first quartile), media, Q75 (third quartile).

SI.2.1. Agronomic properties

Data expressed as follows: ^1^ no unit, ^2^ in gram per kilogram of dry matter, ^3^ in % of fresh matter, ^4^ in microsiever per centimeter.

| **Variable** | **Origin** | **% detection** | **Min** | **Mean** | **Max** | **SD** | **Q25** | **Median** | **Q75** |
| --- | --- | --- | --- | --- | --- | --- | --- | --- | --- |
| C/N^1^ | Crops/residues | 70 % | 2.8 | 7.0 | 13.6 | 3.3 | 4.8 | 6.2 | 8.8 |
|  | Manure | 68 % | 2.9 | 11.4 | 34.3 | 7.1 | 6.5 | 10.1 | 13.4 |
|  | Industrial | 78.95 % | 3.7 | 14.8 | 38.2 | 10.8 | 6.0 | 13.0 | 18.0 |
|  | Urban | 61.29 % | 0.7 | 9.6 | 19.1 | 4.9 | 5.6 | 10.3 | 13.3 |
|  | Other/mixed | 66.67 % | 3.9 | 42.9 | 388.6 | 100.3 | 4.6 | 8.1 | 17.3 |
| C_org^2^ | Crops/residues | 20 % | 32.4 | 66.2 | 100.0 | 47.8 | 49.3 | 66.2 | 83.1 |
|  | Manure | 36 % | 188.5 | 356.8 | 623.3 | 110.8 | 282.8 | 336.3 | 422.3 |
|  | Industrial | 10.53 % | 108.9 | 276.2 | 443.5 | 236.6 | 192.6 | 276.2 | 359.9 |
|  | Urban | 38.71 % | 161.0 | 249.4 | 362.0 | 60.9 | 209.5 | 247.3 | 298.0 |
|  | Other/mixed | 20 % | 277.0 | 591.8 | 814.5 | 280.4 | 480.5 | 684.0 | 749.3 |
| C_total^2^ | Crops/residues | 60 % | 292.9 | 372.1 | 427.0 | 41.2 | 350.8 | 382.3 | 395.4 |
|  | Manure | 40 % | 231.9 | 357.6 | 454.8 | 51.0 | 340.6 | 362.0 | 382.0 |
|  | Industrial | 10.53 % | 312.2 | 350.4 | 412.8 | 54.5 | 319.2 | 326.1 | 369.5 |
|  | Urban | 38.71 % | 221.0 | 279.3 | 374.9 | 46.5 | 243.3 | 270.0 | 304.5 |
|  | Other/mixed | 40 % | 310.3 | 438.8 | 760.0 | 177.0 | 343.2 | 350.0 | 449.0 |
| Ca_total^2^ | Crops/residues | 30 % | 24.1 | 34.9 | 59.1 | 16.4 | 25.5 | 28.2 | 37.7 |
|  | Manure | 78 % | 6.0 | 31.0 | 134.0 | 22.8 | 18.7 | 25.1 | 36.9 |
|  | Industrial | 78.95 % | 4.1 | 149.7 | 373.4 | 132.4 | 43.2 | 97.4 | 285.9 |
|  | Urban | 48.39 % | 20.2 | 51.7 | 172.7 | 37.3 | 29.2 | 35.0 | 60.1 |
|  | Other/mixed | 53.33 % | 7.1 | 34.3 | 124.0 | 29.7 | 22.9 | 26.6 | 30.6 |
| Dry mass^3^ | Crops/residues | 100 % | 3.0 | 16.2 | 50.0 | 14.9 | 5.5 | 9.7 | 21.2 |
|  | Manure | 96 % | 2.0 | 28.3 | 97.5 | 20.2 | 9.3 | 26.2 | 36.9 |
|  | Industrial | 94.74 % | 3.1 | 36.2 | 98.9 | 26.4 | 16.1 | 31.2 | 53.0 |
|  | Urban | 93.55 % | 1.1 | 41.6 | 94.3 | 25.1 | 19.7 | 45.9 | 60.9 |
|  | Other/mixed | 86.67 % | 5.7 | 30.8 | 92.9 | 28.2 | 7.5 | 25.3 | 46.0 |
| Electrical | Manure | 2 % | 6854.0 | 6854.0 | 6854.0 |  | 6854.0 | 6854.0 | 6854.0 |
| conductivity^4^ | Urban | 6.45 % | 1131.0 | 1776.5 | 2422.0 | 912.9 | 1453.8 | 1776.5 | 2099.3 |
|  | Other/mixed | 20 % | 2947.1 | 6604.2 | 10297.0 | 3004.2 | 5542.8 | 6586.3 | 7647.8 |
| K_total^2^ | Crops/residues | 90 % | 2.6 | 43.6 | 94.6 | 32.2 | 13.1 | 42.7 | 72.3 |
|  | Manure | 88 % | 9.3 | 41.3 | 266.0 | 41.6 | 21.9 | 30.6 | 45.7 |
|  | Industrial | 89.47 % | 0.2 | 14.3 | 82.8 | 26.9 | 0.6 | 1.2 | 9.0 |
|  | Urban | 87.1 % | 1.2 | 16.9 | 154.5 | 28.3 | 4.6 | 8.6 | 12.8 |
|  | Other/mixed | 93.33 % | 2.6 | 31.9 | 80.2 | 23.6 | 14.4 | 28.0 | 50.9 |
| Mg_total^2^ | Crops/residues | 30 % | 4.6 | 11.9 | 26.9 | 10.2 | 5.7 | 8.0 | 14.1 |
|  | Manure | 80 % | 2.3 | 8.3 | 58.0 | 8.5 | 5.0 | 6.4 | 8.1 |
|  | Industrial | 89.47 % | 0.3 | 8.3 | 38.5 | 9.1 | 3.3 | 6.3 | 9.5 |
|  | Urban | 48.39 % | 1.7 | 7.1 | 29.5 | 7.8 | 3.0 | 4.4 | 5.8 |
|  | Other/mixed | 53.33 % | 1.6 | 6.9 | 17.5 | 4.1 | 3.9 | 6.6 | 7.8 |
| NH4/N^1^ | Crops/residues | 60 % | 0.1 | 0.4 | 0.6 | 0.2 | 0.3 | 0.4 | 0.6 |
|  | Manure | 54 % | 0.0 | 0.3 | 0.7 | 0.2 | 0.2 | 0.3 | 0.4 |
|  | Industrial | 10.53 % | 0.3 | 0.5 | 0.6 | 0.1 | 0.4 | 0.6 | 0.6 |
|  | Urban | 22.58 % | 0.0 | 0.3 | 0.6 | 0.2 | 0.1 | 0.4 | 0.5 |
|  | Other/mixed | 26.67 % | 0.1 | 0.3 | 0.5 | 0.2 | 0.2 | 0.3 | 0.5 |
| N_NH4^2^ | Crops/residues | 90 % | 0.0 | 39.1 | 82.7 | 30.1 | 8.2 | 39.6 | 67.4 |
|  | Manure | 82 % | 0.1 | 18.3 | 112.1 | 22.4 | 3.7 | 8.8 | 26.1 |
|  | Industrial | 73.68 % | 0.0 | 12.1 | 104.2 | 28.7 | 0.1 | 0.2 | 4.7 |
|  | Urban | 87.1 % | 0.0 | 25.7 | 463.6 | 76.6 | 1.2 | 4.8 | 18.6 |
|  | Other/mixed | 73.33 % | 0.9 | 22.1 | 67.8 | 20.6 | 5.5 | 17.8 | 30.6 |
| N_NO3^2^ | Manure | 8 % | 0.0 | 0.3 | 1.2 | 0.5 | 0.1 | 0.1 | 0.2 |
|  | Industrial | 52.63 % | 0.0 | 0.1 | 0.3 | 0.1 | 0.0 | 0.0 | 0.1 |
|  | Urban | 19.35 % | 0.1 | 0.2 | 0.4 | 0.1 | 0.2 | 0.2 | 0.2 |
|  | Other/mixed | 26.67 % | 0.0 | 1.2 | 5.7 | 2.5 | 0.0 | 0.1 | 0.2 |
| N_org^2^ | Crops/residues | 60 % | 25.5 | 38.1 | 64.0 | 12.0 | 31.6 | 35.0 | 39.0 |
|  | Manure | 70 % | 8.0 | 24.2 | 46.0 | 8.9 | 19.0 | 24.1 | 26.7 |
|  | Industrial | 63.16 % | 0.8 | 20.3 | 56.0 | 19.8 | 3.6 | 15.2 | 28.9 |
|  | Urban | 48.39 % | 8.0 | 23.0 | 56.2 | 14.7 | 12.2 | 15.6 | 35.0 |
|  | Other/mixed | 53.33 % | 7.6 | 28.7 | 42.8 | 12.2 | 18.8 | 31.8 | 39.5 |
| N_total^2^ | Crops/residues | 100 % | 7.2 | 64.8 | 160.0 | 44.6 | 31.7 | 60.6 | 77.1 |
|  | Manure | 94 % | 8.0 | 47.8 | 325.0 | 46.1 | 25.6 | 33.0 | 54.1 |
|  | Industrial | 94.74 % | 0.8 | 25.7 | 133.3 | 35.3 | 4.7 | 9.4 | 27.8 |
|  | Urban | 90.32 % | 6.7 | 52.6 | 536.4 | 86.8 | 16.5 | 22.7 | 55.8 |
|  | Other/mixed | 100 % | 1.8 | 44.4 | 105.0 | 36.5 | 14.8 | 24.4 | 71.4 |
| Na_total^2^ | Crops/residues | 40 % | 2.4 | 5.3 | 8.6 | 2.9 | 3.5 | 3.8 | 8.4 |
|  | Manure | 52 % | 0.5 | 6.8 | 37.0 | 7.5 | 3.4 | 4.7 | 7.0 |
|  | Industrial | 10.53 % | 3.7 | 7.9 | 12.1 | 5.9 | 5.8 | 7.9 | 10.0 |
|  | Urban | 19.35 % | 1.4 | 4.9 | 15.3 | 5.3 | 1.8 | 3.2 | 4.6 |
|  | Other/mixed | 40 % | 0.1 | 9.1 | 34.6 | 12.1 | 1.5 | 4.6 | 10.0 |
| Organic | Crop/residues | 100.0 % | 103 | 603 | 800 | 197 | 552 | 659 | 724 |
| matter^2^ | Industrial | 78.9 % | 129 | 433 | 887 | 257 | 181 | 420 | 622 |
|  | Manures | 90.0 % | 250 | 691 | 912 | 129 | 627 | 686 | 779 |
|  | Other/mixed | 75.0 % | 44 | 572 | 771 | 178 | 564 | 621 | 651 |
|  | Urban | 87.1 % | 299 | 522 | 780 | 120 | 428 | 501 | 616 |
| P_available^2^ | Crops/residues | 30 % | 2.1 | 4.7 | 7.3 | 2.6 | 3.3 | 4.6 | 5.9 |
|  | Manure | 6 % | 0.1 | 1.5 | 2.7 | 1.3 | 0.8 | 1.6 | 2.2 |
|  | Industrial | 10.53 % | 0.0 | 0.0 | 0.0 | 0.0 | 0.0 | 0.0 | 0.0 |
|  | Urban | 32.26 % | 0.0 | 0.9 | 3.3 | 1.1 | 0.2 | 0.4 | 1.6 |
|  | Other/mixed | 26.67 % | 0.1 | 0.9 | 2.2 | 1.0 | 0.1 | 0.6 | 1.4 |
| P_total^2^ | Crops/residues | 90 % | 1.6 | 14.7 | 47.5 | 12.2 | 8.9 | 9.9 | 17.5 |
|  | Manure | 94 % | 2.0 | 11.4 | 41.0 | 7.1 | 6.9 | 10.0 | 14.6 |
|  | Industrial | 94.74 % | 0.5 | 10.3 | 42.2 | 12.6 | 1.6 | 4.0 | 17.1 |
|  | Urban | 90.32 % | 1.4 | 12.4 | 36.2 | 9.7 | 4.2 | 10.5 | 16.8 |
|  | Other/mixed | 93.33 % | 0.3 | 15.4 | 34.0 | 9.6 | 8.7 | 15.8 | 19.9 |
| S_total^2^ | Crops/residues | 20 % | 2.7 | 3.6 | 4.5 | 1.3 | 3.1 | 3.6 | 4.0 |
|  | Manure | 50 % | 0.6 | 9.0 | 39.0 | 10.7 | 3.8 | 5.3 | 8.0 |
|  | Industrial | 10.53 % | 5.6 | 9.9 | 15.0 | 4.8 | 7.3 | 9.0 | 12.0 |
|  | Urban | 9.68 % | 5.0 | 5.9 | 7.0 | 1.0 | 5.3 | 5.6 | 6.3 |
|  | Other/mixed | 40 % | 0.2 | 5.8 | 8.4 | 2.6 | 5.3 | 6.4 | 7.2 |
| pH | Crops/residues | 70 % | 7.3 | 8.0 | 8.7 | 0.4 | 7.9 | 8.0 | 8.2 |
|  | Manure | 66 % | 5.9 | 8.2 | 9.4 | 0.7 | 7.9 | 8.1 | 8.6 |
|  | Industrial | 89.47 % | 6.0 | 8.6 | 12.8 | 1.9 | 7.5 | 8.1 | 9.5 |
|  | Urban | 83.87 % | 6.8 | 8.3 | 12.5 | 1.2 | 7.7 | 8.1 | 8.5 |
|  | Other/mixed | 86.67 % | 7.3 | 8.5 | 10.6 | 0.8 | 8.1 | 8.3 | 8.7 |

SI.2.2. Trace metals

Data expressed in milligram per kilogram of dry matter.

| **Variable** | **Origin** | **% detection** | **Min** | **Mean** | **Max** | **SD** | **Q25** | **Median** | **Q75** |
| --- | --- | --- | --- | --- | --- | --- | --- | --- | --- |
| Ag_total | Manure | 4 % | 0.0 | 0.0 | 0.1 | 0.0 | 0.0 | 0.0 | 0.1 |
|  | Urban | 9.68 % | 0.3 | 4.1 | 7.6 | 3.7 | 2.4 | 4.5 | 6.0 |
| Al_total | Manure | 12 % | 0.6 | 2.5 | 6.6 | 2.7 | 0.7 | 1.0 | 4.3 |
|  | Urban | 19.35 % | 2.0 | 18.9 | 33.5 | 10.6 | 14.8 | 19.9 | 23.6 |
| As_total | Crops/residues | 40 % | 0.8 | 1.8 | 3.4 | 1.2 | 0.9 | 1.2 | 2.9 |
|  | Manure | 40 % | 0.0 | 1.0 | 4.4 | 1.0 | 0.3 | 0.7 | 1.5 |
|  | Industrial | 68.42 % | 0.1 | 1.9 | 4.9 | 1.3 | 1.2 | 1.8 | 2.1 |
|  | Urban | 48.39 % | 2.1 | 5.5 | 12.7 | 2.9 | 2.8 | 5.0 | 7.4 |
|  | Other/mixed | 46.67 % | 0.0 | 2.5 | 8.4 | 3.3 | 0.7 | 0.9 | 2.1 |
| B_total | Manure | 20 % | 1.7 | 13.1 | 44.0 | 15.2 | 7.0 | 7.0 | 8.5 |
|  | Urban | 12.9 % | 1.0 | 2.9 | 4.6 | 1.4 | 2.0 | 3.0 | 3.8 |
| Ba_total | Manure | 10 % | 17.7 | 52.0 | 138.0 | 31.8 | 38.0 | 44.5 | 51.0 |
| Cd_total | Crops/residues | 40 % | 0.3 | 0.5 | 0.7 | 0.1 | 0.5 | 0.5 | 0.6 |
|  | Manure | 52 % | 0.0 | 0.4 | 0.9 | 0.2 | 0.2 | 0.4 | 0.5 |
|  | Industrial | 68.42 % | 0.1 | 0.6 | 1.8 | 0.5 | 0.2 | 0.5 | 0.9 |
|  | Urban | 61.29 % | 0.2 | 1.0 | 4.6 | 0.9 | 0.6 | 0.9 | 1.1 |
|  | Other/mixed | 53.33 % | 0.3 | 0.6 | 1.0 | 0.2 | 0.5 | 0.7 | 0.8 |
| Co_total | Crops/residues | 30 % | 1.7 | 2.6 | 3.6 | 0.8 | 2.3 | 2.6 | 2.9 |
|  | Manure | 40 % | 0.8 | 2.7 | 6.5 | 1.5 | 1.5 | 2.4 | 3.6 |
|  | Industrial | 21.05 % | 1.0 | 1.9 | 3.1 | 0.9 | 1.1 | 1.5 | 2.6 |
|  | Urban | 32.26 % | 1.4 | 10.0 | 18.4 | 5.0 | 7.1 | 11.4 | 12.3 |
|  | Other/mixed | 20 % | 0.1 | 2.5 | 4.8 | 2.1 | 1.3 | 2.6 | 3.9 |
| CrVI_total | Other/mixed | 6.67 % | 0.4 | 0.4 | 0.4 |  | 0.4 | 0.4 | 0.4 |
| Cr_total | Crops/residues | 40 % | 2.1 | 15.5 | 26.1 | 8.8 | 13.9 | 16.6 | 19.0 |
|  | Manure | 54 % | 0.3 | 7.8 | 24.7 | 5.4 | 4.7 | 6.2 | 9.6 |
|  | Industrial | 68.42 % | 1.7 | 31.6 | 268.0 | 68.6 | 8.7 | 12.3 | 20.4 |
|  | Urban | 61.29 % | 3.2 | 35.0 | 126.3 | 25.7 | 18.7 | 30.0 | 37.5 |
|  | Other/mixed | 46.67 % | 4.2 | 25.7 | 75.0 | 19.7 | 14.5 | 17.0 | 28.9 |
| Cu_total | Crops/residues | 40 % | 57.2 | 130.6 | 217.6 | 59.2 | 105.7 | 122.7 | 149.7 |
|  | Manure | 66 % | 2.0 | 99.5 | 486.0 | 102.9 | 33.3 | 71.8 | 120.0 |
|  | Industrial | 68.42 % | 9.2 | 45.2 | 158.6 | 42.2 | 22.6 | 27.4 | 52.4 |
|  | Urban | 61.29 % | 12.0 | 134.8 | 384.7 | 122.3 | 46.1 | 82.9 | 186.3 |
|  | Other/mixed | 60 % | 9.7 | 78.7 | 172.6 | 55.8 | 25.5 | 72.0 | 117.5 |
| Fe_total | Manure | 18 % | 1.0 | 3.0 | 5.9 | 1.5 | 2.3 | 2.7 | 3.4 |
|  | Urban | 16.13 % | 8.8 | 17.4 | 28.6 | 9.4 | 9.1 | 14.4 | 26.3 |
| Hg_total | Crops/residues | 20 % | 0.0 | 0.3 | 0.6 | 0.4 | 0.2 | 0.3 | 0.4 |
|  | Manure | 30 % | 0.0 | 0.2 | 2.7 | 0.5 | 0.0 | 0.1 | 0.1 |
|  | Industrial | 68.42 % | 0.0 | 0.2 | 1.2 | 0.4 | 0.0 | 0.1 | 0.1 |
|  | Urban | 54.84 % | 0.0 | 0.4 | 1.9 | 0.5 | 0.1 | 0.2 | 0.6 |
|  | Other/mixed | 46.67 % | 0.0 | 0.2 | 0.4 | 0.1 | 0.1 | 0.1 | 0.2 |
| Li_total | Manure | 4 % | 5.8 | 6.8 | 7.8 | 1.0 | 6.4 | 6.9 | 7.4 |
| Mn_total | Manure | 34 % | 60.0 | 866.1 | 6398.5 | 1333.5 | 121.0 | 520.0 | 963.0 |
|  | Urban | 22.58 % | 29.0 | 255.1 | 625.9 | 212.6 | 101.9 | 212.5 | 361.4 |
| Mo_total | Crops/residues | 30 % | 3.2 | 6.6 | 11.8 | 3.7 | 4.7 | 5.7 | 7.6 |
|  | Manure | 46 % | 1.2 | 4.3 | 10.7 | 2.6 | 2.6 | 3.1 | 5.9 |
|  | Industrial | 10.53 % | 0.9 | 3.2 | 6.6 | 3.0 | 1.6 | 2.2 | 4.4 |
|  | Urban | 29.03 % | 1.0 | 5.0 | 10.0 | 3.1 | 2.7 | 4.5 | 7.9 |
|  | Other/mixed | 13.33 % | 1.8 | 3.4 | 4.8 | 1.5 | 2.7 | 3.5 | 4.2 |
| Ni_total | Crops/residues | 40 % | 9.4 | 12.4 | 15.4 | 2.2 | 11.9 | 11.9 | 13.2 |
|  | Manure | 54 % | 0.7 | 15.8 | 154.6 | 22.8 | 6.2 | 9.5 | 17.4 |
|  | Industrial | 68.42 % | 4.6 | 13.1 | 33.0 | 7.6 | 6.6 | 12.5 | 15.8 |
|  | Urban | 61.29 % | 3.2 | 20.7 | 60.4 | 11.4 | 12.3 | 20.4 | 25.5 |
|  | Other/mixed | 60 % | 5.9 | 13.2 | 22.5 | 5.2 | 9.2 | 13.5 | 15.9 |
| Pb_total | Crops/residues | 40 % | 7.4 | 9.0 | 12.0 | 1.7 | 8.4 | 8.5 | 8.7 |
|  | Manure | 54 % | 0.1 | 4.5 | 34.2 | 6.4 | 0.9 | 2.6 | 4.9 |
|  | Industrial | 68.42 % | 1.0 | 7.6 | 18.8 | 6.0 | 3.1 | 5.5 | 13.4 |
|  | Urban | 61.29 % | 2.5 | 59.4 | 352.9 | 62.6 | 32.1 | 44.9 | 63.5 |
|  | Other/mixed | 60 % | 1.5 | 12.9 | 35.8 | 10.1 | 8.2 | 10.9 | 13.1 |
| Se_total | Crops/residues | 20 % | 0.5 | 4.4 | 8.3 | 5.5 | 2.5 | 4.4 | 6.4 |
|  | Manure | 30 % | 0.1 | 5.1 | 28.3 | 7.5 | 0.5 | 1.8 | 6.5 |
|  | Industrial | 10.53 % | 2.2 | 3.9 | 4.8 | 1.5 | 3.5 | 4.8 | 4.8 |
|  | Urban | 32.26 % | 0.0 | 1.3 | 3.0 | 1.0 | 0.6 | 0.9 | 2.0 |
|  | Other/mixed | 6.67 % | 56.8 | 56.8 | 56.8 |  | 56.8 | 56.8 | 56.8 |
| Sn_total | Manure | 6 % | 1.3 | 1.4 | 1.4 | 0.1 | 1.3 | 1.4 | 1.4 |
| Sr_total | Manure | 10 % | 30.8 | 55.0 | 112.0 | 21.4 | 44.3 | 54.7 | 58.6 |
| Tl_total | Crops/residues | 30 % | 0.3 | 0.4 | 0.9 | 0.3 | 0.3 | 0.3 | 0.5 |
|  | Manure | 10 % | 0.0 | 0.1 | 0.2 | 0.1 | 0.0 | 0.1 | 0.1 |
|  | Urban | 29.03 % | 0.1 | 0.6 | 2.2 | 0.8 | 0.2 | 0.3 | 0.3 |
| V_total | Manure | 10 % | 5.6 | 10.3 | 20.2 | 4.9 | 7.2 | 8.7 | 11.2 |
| Zn_total | Crops/residues | 40 % | 186.3 | 407.1 | 696.6 | 223.6 | 211.5 | 367.7 | 573.6 |
|  | Manure | 66 % | 12.0 | 407.1 | 4050.0 | 600.5 | 142.0 | 203.2 | 400.8 |
|  | Industrial | 68.42 % | 22.8 | 182.8 | 774.7 | 196.1 | 46.2 | 137.0 | 220.7 |
|  | Urban | 61.29 % | 63.0 | 391.7 | 994.9 | 252.3 | 177.4 | 325.7 | 582.6 |
|  | Other/mixed | 60 % | 21.5 | 279.2 | 524.1 | 165.3 | 153.9 | 286.8 | 393.4 |

SI.2.3. Organic contaminants

Data expressed in ^1^ milligram per kilogram of dry matter and ^2^ microgram per kilogram of dry matter.

| **Variable** | **Origin** | **% detection** | **Min** | **Mean** | **Max** | **SD** | **Q25** | **Median** | **Q75** |
| --- | --- | --- | --- | --- | --- | --- | --- | --- | --- |
| 16 PAH_sum^1^ | Manure | 12 % | 0.2 | 3.3 | 16.5 | 5.4 | 0.6 | 1.6 | 2.4 |
|  | Industrial | 52.63 % | 0.1 | 0.6 | 1.9 | 0.5 | 0.2 | 0.3 | 0.8 |
|  | Urban | 38.71 % | 0.9 | 3.5 | 16.9 | 4.3 | 1.5 | 2.5 | 3.3 |
|  | Other/mixed | 26.67 % | 0.5 | 1.3 | 2.3 | 0.7 | 1.0 | 1.0 | 1.9 |
| 7 PCB_sum^1^ | Manure | 10 % | 0.0 | 0.0 | 0.1 | 0.0 | 0.0 | 0.0 | 0.1 |
|  | Industrial | 52.63 % | 0.0 | 0.2 | 1.5 | 0.5 | 0.0 | 0.0 | 0.1 |
|  | Urban | 45.16 % | 0.0 | 0.1 | 0.4 | 0.1 | 0.0 | 0.0 | 0.1 |
|  | Other/mixed | 20 % | 0.0 | 0.1 | 0.1 | 0.0 | 0.0 | 0.1 | 0.1 |
| ATB_fluoroquinolones_sum^2^ | Manure | 6 % | 9.2 | 117.6 | 327.0 | 181.4 | 12.9 | 16.6 | 171.8 |
|  | Urban | 16.13 % | 29.2 | 4603.1 | 13183.0 | 5775.5 | 29.6 | 1902.5 | 7871.0 |
| ATB_macrolides_sum^2^ | Manure | 2 % | 149.0 | 149.0 | 149.0 |  | 149.0 | 149.0 | 149.0 |
| ATB_sulflonamides_sum^2^ | Manure | 2 % | 21.1 | 21.1 | 21.1 |  | 21.1 | 21.1 | 21.1 |
| ATB_tetracyclines_sum^2^ | Manure | 8 % | 1421.0 | 8888.3 | 30005.0 | 14082.6 | 1713.5 | 2063.5 | 9238.3 |
|  | Urban | 3.23 % | 15054.0 | 15054.0 | 15054.0 |  | 15054.0 | 15054.0 | 15054.0 |
| Anti-inflammatories_sum^2^ | Manure | 2 % | 23.0 | 23.0 | 23.0 |  | 23.0 | 23.0 | 23.0 |
|  | Urban | 16.13 % | 66.7 | 521.8 | 1908.0 | 779.4 | 122.7 | 246.0 | 265.4 |
| BTEX_total^1^ | Manure | 4 % | 0.2 | 0.7 | 1.1 | 0.4 | 0.5 | 0.8 | 1.0 |
|  | Industrial | 52.63 % | 0.1 | 0.3 | 0.8 | 0.3 | 0.1 | 0.2 | 0.3 |
|  | Urban | 12.9 % | 0.1 | 8.9 | 43.3 | 19.2 | 0.2 | 0.3 | 0.5 |
|  | Other/mixed | 20 % | 0.2 | 0.6 | 1.0 | 0.4 | 0.2 | 0.6 | 0.9 |
| Benzo(a)pyren^2^ | Urban | 19.35 % | 90.0 | 183.3 | 250.0 | 75.6 | 119.6 | 200.0 | 250.0 |
|  | Other/mixed | 6.67 % | 0.0 | 0.0 | 0.0 |  | 0.0 | 0.0 | 0.0 |
| Benzo(b)fluoranthen^2^ | Urban | 19.35 % | 114.0 | 339.0 | 500.0 | 180.8 | 195.0 | 370.0 | 500.0 |
|  | Other/mixed | 6.67 % | 0.0 | 0.0 | 0.0 |  | 0.0 | 0.0 | 0.0 |
| Dioxin_sum^1^ | Manure | 2 % | 0.0 | 0.0 | 0.0 |  | 0.0 | 0.0 | 0.0 |
|  | Other/mixed | 6.67 % | 0.9 | 0.9 | 0.9 |  | 0.9 | 0.9 | 0.9 |
| Fluoranthen^1^ | Crops/residues | 30 % | 0.0 | 0.1 | 0.1 | 0.0 | 0.0 | 0.0 | 0.1 |
|  | Manure | 10 % | 0.0 | 0.1 | 0.8 | 0.3 | 0.0 | 0.0 | 0.0 |
|  | Industrial | 5.26 % | 0.0 | 0.0 | 0.0 |  | 0.0 | 0.0 | 0.0 |
|  | Urban | 38.71 % | 0.0 | 0.5 | 0.9 | 0.3 | 0.2 | 0.5 | 0.8 |
|  | Other/mixed | 6.67 % | 0.0 | 0.0 | 0.0 |  | 0.0 | 0.0 | 0.0 |
| Halogenated solvents^1^ | Manure | 2 % | 0.7 | 0.7 | 0.7 |  | 0.7 | 0.7 | 0.7 |
|  | Industrial | 21.05 % | 0.2 | 69.6 | 295.0 | 120.0 | 0.4 | 2.0 | 89.5 |
|  | Urban | 12.9 % | 0.1 | 1.7 | 5.5 | 2.5 | 0.3 | 0.6 | 1.9 |
|  | Other/mixed | 20 % | 0.3 | 76.2 | 301.6 | 150.2 | 0.6 | 1.6 | 77.2 |
| NEP_sum^2^ | Manure | 6 % | 1579.0 | 5029.7 | 11680.0 | 5760.7 | 1704.5 | 1830.0 | 6755.0 |
|  | Urban | 6.45 % | 1941.0 | 16772.5 | 31604.0 | 20974.9 | 9356.8 | 16772.5 | 24188.3 |
| PAH_6 Borneff^1^ | Manure | 4 % | 0.2 | 0.8 | 1.3 | 0.6 | 0.6 | 1.0 | 1.2 |
|  | Industrial | 52.63 % | 0.0 | 0.2 | 0.5 | 0.2 | 0.1 | 0.2 | 0.3 |
|  | Urban | 16.13 % | 1.6 | 3.2 | 8.9 | 3.2 | 1.7 | 1.8 | 2.1 |
|  | Other/mixed | 20 % | 0.5 | 1.0 | 1.7 | 0.5 | 0.7 | 0.9 | 1.3 |
| PFAS_sum^2^ | Manure | 8 % | 0.0 | 0.9 | 1.4 | 0.6 | 0.7 | 1.1 | 1.3 |
|  | Urban | 16.13 % | 11.1 | 284.1 | 479.0 | 244.1 | 23.2 | 441.5 | 465.8 |
| anti-epileptic^2^ | Urban | 16.13 % | 2.3 | 44.0 | 108.0 | 42.8 | 17.9 | 26.2 | 65.9 |
| antidepressant^2^ | Urban | 6.45 % | 14.0 | 176.0 | 338.0 | 229.1 | 95.0 | 176.0 | 257.0 |
| bactericide^2^ | Manure | 4 % | 617.0 | 1475.5 | 2334.0 | 1214.1 | 1046.3 | 1475.5 | 1904.8 |
|  | Urban | 12.9 % | 56.0 | 1018.8 | 2895.0 | 1275.5 | 368.0 | 562.2 | 1213.0 |

SI.3. EOM characteristic database format under Zenodo repository


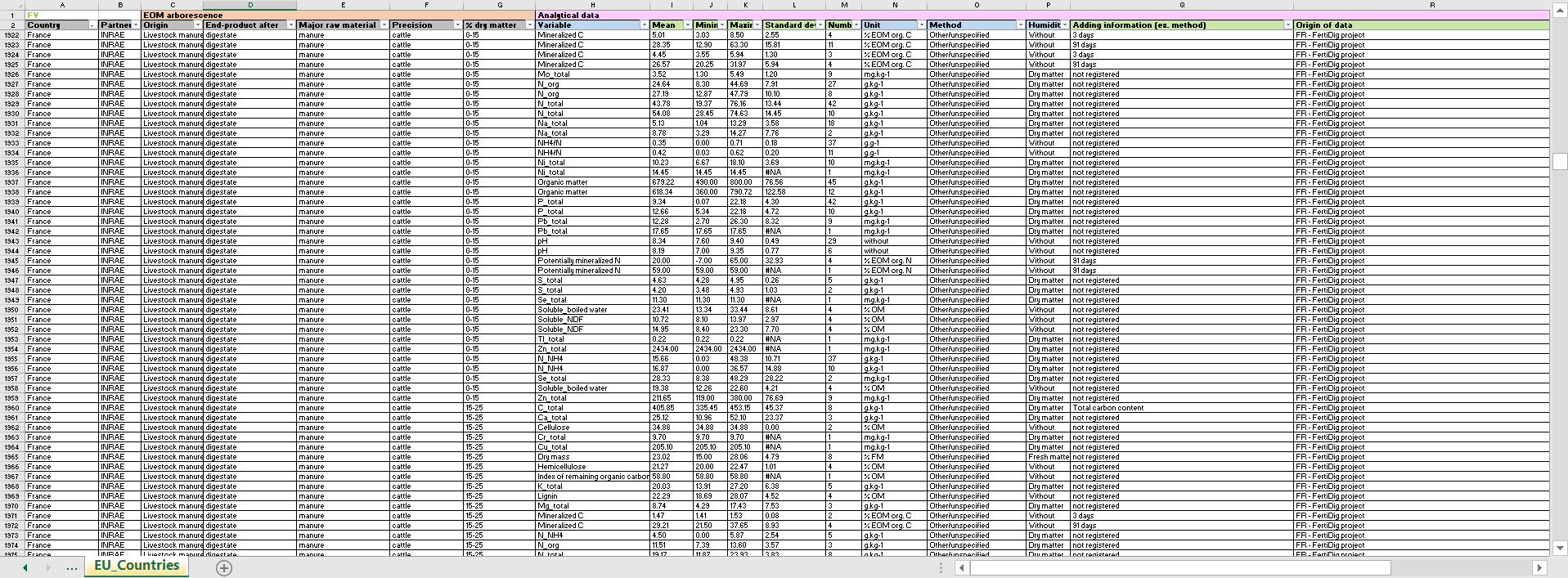
 SI.4. EOM4SOIL project partners and EOM data collection from them


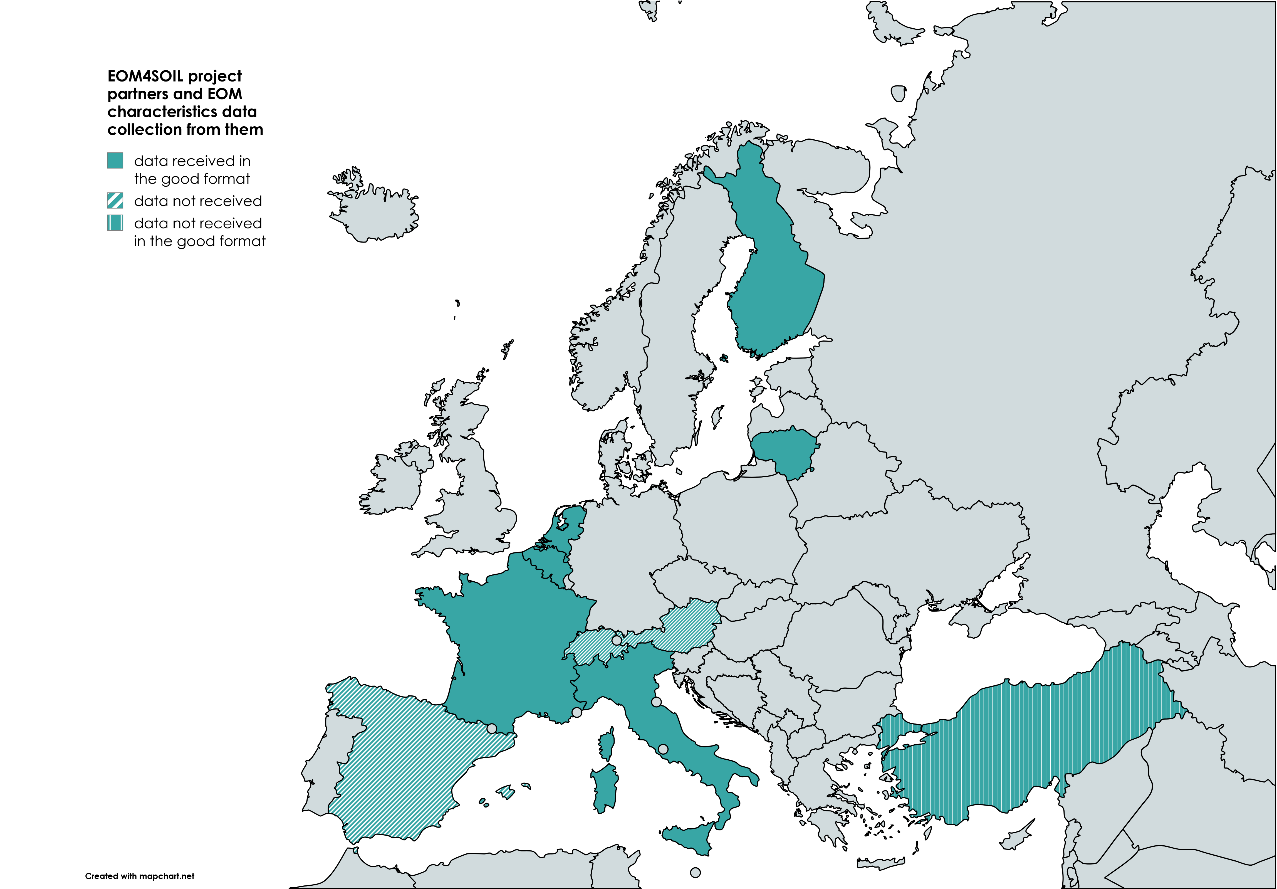


Map generated with MapChart <https://www.mapchart.net/> under licence CC-BY-SA.
